# Notes and News

**Published:** 1901-04-13

**Authors:** 


### Notes and News.

The smallpox epidemic in Glasgow is again abating, and the small outbreak at Cardiff shows no signs of spreading.

Sir William MacCormac is the recipient of an honour from the King of Italy, who has created him a Grand Officer of the Order of the King of Italy.

The Portuguese Government is sending to Angola a commission to study the sleeping sickness peculiar to the district, and also various aspects of malaria.

Sir William Church and Sir Dyce Duckworth are appointed as delegates of the Royal College of Physicians to attend the forthcoming jubilee of the Glasgow University.

In Missouri pressure is being brought to bear against the treatment of sickness by Christian scientists. A Bill is before the Legislature which is framed to prohibit Christian scientists from treating sick persons without a license, for payment.

The experience at the Manchester Consumption Hospital at Bowden adds another testimony to the successful use of the open-air treatment. Since the adoption of this system, the numbers of patients who left the hospital greatly improved have been immensely larger than before.

An International Congress of Life Assurance will be held in September at Amsterdam. The institution of these congresses has been to enable the medical officers of life insurance societies to arrive at a uniform and wise basis of determining the reasons for accepting or rejecting the applicants for life insurance.

An outbreak of three cases of smallpox at Hebburn-on-Tyne, in Northumberland, has been traced to infection in Glasgow.

Although very fully occupied with ceremonials during his stay at Nice, President Loubet devoted a morning to visiting the Civil and Military hospitals.

There are upwards of 120 cases of plague under treatment at Capetown, nearly one-third of the cases being amongst Europeans. The total number of deaths since the outbreak rather exceeds this number. A case of plague is reported from Alexandria.

Dr. E. Symes Thompson will lecture on April 16th, 17th, 18th and 19th, at 6 p.m., on the Climate of Algiers at the Gresham College, Basinghall Street. Many invalids are now seeking Algiers as a health resort in preference to the Riviera, but far less is known generally as to its climate.

The German Medical Schools are required again to consider if a rigid adherence to a classical education is necessary before students are admitted to enter for their medical studies. The objection to any alteration is, of course, that it is considered that less educated persons would gain admission to the medical profession, and thus lower the status of medical men generally.

In the attempt to justify opinions some people are carried very far. This was surely the case with Dr. von Koeber, President of the Anti-Alcohol Congress, held in Vienna, at which he stated that 50 per cent. of the Austrian children were accustomed to taking strong drink; and with Dr. Meinhert, who stated that mortality amongst medical men was exceptionally high owing to their indulgence in strong drink and morphine.

At a meeting of the Special Election Committee of the Liverpool Royal Infirmary, held on the 3rd instant, Dr Thomas R. Bradshaw, M.D., M.R.C.P.Lond., who had been one of the honorary assistant physicians to the institution since the year 1892, was unanimously elected to fill the vacancy on the senior staff created by Dr. Glynn's retirement. A subsequent special meeting of trustees altered the laws so as to constitute the surgeons of the Lock Branch members of the Medical Board.

The active and important part taken by rats in the spread of disease, and more especially of the plague, is now so fully recognised that the London County Council have recommended the authorities directly concerned with wharves and riverside premises to study the subject of the arrival of rats on shipping. Rats are also said to be responsible for the spread of disease amongst cattle. An epidemic of foot and mouth disease in Suffolk is put down to their agency. No less than 500 cattle have had to be destroyed owing to this epidemic.

